# Peroxidase expression is decreased by palmitate in cultured podocytes but increased in podocytes of advanced diabetic nephropathy

**DOI:** 10.1002/jcp.26875

**Published:** 2018-08-21

**Authors:** Eugene Lee, Hyun Soon Lee

**Affiliations:** ^1^ Renal Pathology Lab, Hankook Kidney and Diabetes Institute Seoul Korea

**Keywords:** diabetic nephropathy, free fatty acids, H_2_O_2_, peroxidase, podocytes

## Abstract

High levels of serum free fatty acids (FFAs) are associated with lipotoxicity and type 2 diabetes. Palmitic acid (PA) is the predominant circulating saturated FFA. PA induces mitochondrial superoxide and hydrogen peroxide (H_2_O _2_) generation in cultured podocytes. To elucidate the role of PA in antioxidant defense systems in diabetic nephropathy (DN), cultured podocytes were exposed to 250 μM PA for 1–24 hr, and protein expressions of catalase, peroxiredoxins (Prxs), and glutathione peroxidase (GPx) were examined by western blot analysis. PA induced an early transient increase in the Prx1, Prx2, and GPx1 levels in podocytes, but not catalase. Long‐term exposure of PA to podocytes significantly decreased the protein levels of Prx1, Prx2, GPx1, and catalase. Coincubation of PA‐treated cells with oleic acid, however, restored the expression of these proteins. In advanced human diabetic glomeruli, H_2_O_2_ generation was elevated as shown by increased fluorescence of dichlorofluorescein. Strong immunostaining for Prx1, Prx2, GPx1, and catalase was observed in the podocytes of advanced human DN, wherein transforming growth factor‐β1 staining was also positive. These results suggest that podocytes are susceptible to PA‐induced oxidative damage with impaired peroxidase activity and that peroxidases have futile antioxidant effects in the podocytes in the late stages of DN. Given this, PA‐induced podocyte injury via inadequate peroxidase response to H_2_O_2_ appears to play an important role in the pathogenesis of DN.

## 1 INTRODUCTION

Diabetic nephropathy (DN) is the leading cause of end‐stage renal disease worldwide (Molitch et al., 2015). Podocyte damage plays a crucial role in the pathogenesis of DN (H. S. Lee, 2013; Wolf, Chen, & Ziyadeh, 2005).

Excess carbohydrate, which cannot be converted into glycogen, is converted into triglyceride (TG) and stored in adipose tissue. In obesity and type 2 diabetes, chronically high levels of serum TG and free fatty acids (FFAs) bring about intracellular fatty acid accumulation in many nonadipose tissues leading to lipotoxicity (H. S. Lee, 2011; Shulman, 2014; Unger, Clark, Scherer, & Orci, 2010). Palmitic acid (PA) is the predominant circulating saturated FFA. PA induces mitochondrial superoxide and hydrogen peroxide (H_2_O_2_) generation in cultured podocytes, while oleic acid (OA) inhibits the PA‐induced reactive oxygen species (ROS) formation (E. Lee, Choi, & Lee, 2017).

In response to excess ROS generation, mammalian cells have evolved a various set of peroxidases that catalyze the conversion of the intracellular H_2_O_2_ to water. They include catalase, peroxiredoxins (Prxs), and glutathione peroxidases (GPxs).

Catalase is a key antioxidant enzyme in the protection of cells against oxidative injury (Meilhac, Zhou, Santanam, & Parthasarathy, 2000; Santanam, Auge, Zhou, Keshava, & Parthasarathy, 1999). Catalase overexpression in the renal proximal tubular epithelial cells in diabetic animals attenuated ROS generation or the progression of nephropathy (Brezniceanu et al., 2007; Shi et al., 2013). PA increased catalase expression as a line of defense against peroxides in cultured tubular epithelial cells (Ruggiero et al., 2014). Incubation of puromycin aminonucleoside in cultured podocytes increased catalase activity (Elvin et al., 2016; Vega‐Warner, Ransom, Vincent, Brosius, & Smoyer, 2004), preceded by enhanced intracellular ROS production (Vega‐Warner et al., 2004). In contrast, long‐term incubation of high glucose (HG) inhibited the podocyte catalase protein expression despite an increase in intracellular ROS generation (Piwkowska, Rogacka, Audzeyenka, Jankowski, & Angielski, 2011).

Prxs are highly abundant ubiquitous antioxidant enzymes (Kang, Rhee, Chang, Jeong, & Choi, 2005; Rhee & Woo, 2011). Prx2 reacts with H_2_O_2_ as fast as catalase (Peskin et al., 2007). Expression of Prx1, Prx3, and Prx5 was increased in the glomeruli of diabetic mice (Barati et al., 2007). Knockdown of Prx2, but not Prx1, induced significant increases in the intracellular ROS in cultured podocytes (Hsu et al., 2011). Transfection of tubular epithelial cells with Prx2 was protective and mitigated apoptosis (Ruggiero et al., 2014). PA led to a decreased expression of Prx2 protein in cultured tubular epithelial cells (Ruggiero et al., 2014), and so did angiotensin II in cultured podocytes (Hsu et al., 2011).

Long‐term exposure of PA in insulinoma MIN6 cells significantly decreased Prx1, Prx2, and Prx4 expression, while OA restored the expression of these proteins (Sargsyan, Artemenko, Manukyan, Bergquist, & Bergsten, 2016).

Of the five GPx isoforms, GPx1 is the only cytosolic enzyme and is mainly present in normal kidneys (de Haan et al., 2005). GP x 1 knockdown or podocyte GPx1 loss in diabetic mice had no increased risk for glomerular damage or oxidative stress as compared with wild‐type diabetic mice (Blauwkamp et al., 2008; de Haan et al., 2005). In this regard, GPx1 may not be protective against oxidative renal injury during the development of DN. In cultured podocytes, HG induced no changes in GPx protein expression (Piwkowska et al., 2011).

It is proposed that excess generation of mitochondrial ROS in response to HG (Nishikawa et al., 2000) or PA (E. Lee et al., 2017) plays a central role in the initiation of DN. Indeed, mitochondrial ROS was increased in the glomeruli of living diabetic mice (Galvan et al., 2017), while Dugan et al. (2013) reported opposite findings.

Little is known about the role of peroxidases in PA‐induced podocyte injury and pathogenesis of DN. In this regard, we examined the expression of catalase, Prx1, Prx2, and GPx1 proteins in cultured podocytes exposed to PA and OA. Furthermore, H_2_O_2_ production and peroxidase expression were examined in the glomeruli of patients with DN.

## 2 MATERIALS AND METHODS

### 2.1 Reagents

Type 1 collagen, fetal calf serum (FCS), and penicillin–streptomycin were from Gibco by Life Technologies (Grand Island, NY). Rabbit polyclonal antibodies for Prx1 (cat. #PA5‐27487, RRID: AB_2544963), GPx1 (cat. #PA5‐27148, RRID: AB_2543823), and β‐actin (cat. #MA5‐15739, RRID: AB_10979409) were from Invitrogen by Thermo Fisher Scientific (Rockford, IL). Mouse monoclonal antibodies for catalase (cat. #LF‐MA0010, RRID: AB_1611843) and Prx2 (cat. #LF‐MA0144, RRID: AB_1620974) were from AbFrontier (Seoul, Korea). Horseradish peroxidase (HRP)–conjugated secondary antibody for rabbit (cat. #7074, RRID: AB_2099233) was from Cell Signaling Technology (Beverly, MA), and HRP‐linked goat anti‐mouse immunoglobulin G (IgG; cat. #LF‐SA800) was from AbFrontier. Rabbit polyclonal antibody for transforming growth factor‐β1 (TGF‐β1; cat. #sc‐146, RRID: AB‐632486) was from Santa Cruz Biotechnology (Dallas, TX). Biotinylated goat anti‐rabbit IgG (code #E0432) and streptavidin‐conjugated HRP complex (code #P0397) were from Dako (Glostrup, Denmark). Biotinylated goat anti‐mouse IgG (cat. #31803, RRID: AB_228311) was from Thermo Fisher Scientific (Rockford, IL). Carboxy‐2′,7′‐dichlorodihydrofluorescein (DCFH) diacetate diacetoxymethyl ester (carboxy‐DCFH DA‐AM) were from Life Technologies (Eugene, OR). PA, OA, and all other chemicals were from Sigma‐Aldrich (St. Louis, MO).

### 2.2 Cell culture

Conditionally immortalized mouse podocytes, kindly provided by Peter Mundel, were cultured as described previously (Mundel et al., 1997). The cells were grown in RPMI 1640 medium supplemented with 10% FCS and 1% penicillin–streptomycin at 33°C in 5% CO_2_–95% air. To induce differentiation, podocytes were maintained at 37°C for 10–14 days.

### 2.3 Fatty acid preparation

Lipid‐containing media were prepared by conjugation of FFAs with bovine serum albumin (BSA) as described previously (E. Lee et al., 2017; Schmitz‐Peiffer, Craig, & Biden, 1999). In brief, 20% (wt/vol) BSA was heated to 37°C before the addition of PA or OA dissolved in ethanol. The solution was heated to 37°C until clear and diluted with RPMI to give a final concentration of 5% BSA, 250 μM PA or OA, and 1% ethanol. The solutions were filter sterilized before being added onto the cells. Control media prepared similarly contained ethanol and BSA in the absence of lipid.

### 2.4 Human subjects

Kidney biopsy samples, diagnosed with DN (age of patients >18 years, *N = *12), idiopathic focal segmental glomerulosclerosis (FSGS; *N* = 5), and IgA nephropathy (IgAN; *N* = 5) were obtained for routine diagnostic procedures. Four controls consisted of biopsy samples from patients with asymptomatic hematuria.

### 2.5 Protein extraction and western blot analysis

Differentiated podocytes in collagen‐coated six‐well plates were serum‐starved for 24 hr and treated with 5% BSA, 250 μM PA or OA for 1–24 hr as described previously (E. Lee et al., 2017). The cells were scraped and purified. Protein quantification was determined using the Lowry method. SDS–PAGE was performed, and the protein was transferred onto polyvinylidene fluoride membranes. The membranes were blocked with 5% BSA and incubated overnight with anti‐catalase, anti‐Prx1, anti‐Prx2, and anti‐GPx1. They were then incubated with an HRP‐linked secondary antibody for 2 hr and luminescence was created using an enhanced chemiluminescence kit, before imaging and analysis with a chemiluminescence imaging system (Alliance‐LD2‐87.WL/Auto; Uvitec; Cambridge, UK). Densitometry was performed and processed using the Gen5 software package (Bio‐Tek, Winooski, VT). To assess the equality of protein loading, the membrane was reprobed with anti‐β‐actin.

### 2.6 Catalase activity assay

Podocytes were exposed to PA for 16 hr. Afterwards, podocytes were lysed on ice in 50 mM potassium phosphate, 0.1 mM ethylenediaminetetraacetic acid (EDTA), 0.1% Triton X‐100 (pH 7.8). Extracts were centrifuged at 12,000*g* for 20 min at 4°C and supernatants were used. The initial rate of disappearance of H_2_O_2_ was recorded at a wavelength of 240 nm during 1 min in reaction cell lysates containing 10 mM H_2_O_2_, 50 mM potassium phosphate, 0.1 mM EDTA, pH 7.0 as described by Aebi (1984).

### 2.7 Detection of glomerular H_**2**_O_**2**_ production

Frozen kidney biopsy samples of patients with DN and controls were cut into 6‐µm‐thick sections and placed on a glass slide. Sections were washed with PBS, and incubated with 10 μM carboxy‐DCFH DA‐AM for 2.5 hr at 37°C in a 5% CO_2_ environment. Dichlorofluorescein (DCF) fluorescence was examined (excitation/emission: 490/516 nm) using a Zeiss fluorescence microscope (AXIO Scope A1; Carl Zeiss, Heidenheim, Germany).

### 2.8 Immunohistochemistry

An avidin–biotin–peroxidase procedure was used for antibody localization. Paraffin‐embedded kidney sections (3 µm) were deparaffinized serially. For antigen retrieval, sections were treated with trypsin (Digest‐All 2; Invitrogen) for 30 min at 37°C. Endogenous peroxidase activity was quenched with 10% methanol–H_2_O_2_ solution for 10 min. Sections were then incubated with primary antibodies against Prx1, Prx2, GPx1, and catalase for 1 hr at room temperature. In addition, they were incubated overnight with rabbit anti‐human TGF‐β1 at 4°C. Biotinylated goat anti‐rabbit IgG or anti‐mouse IgG was used as a secondary antibody. Then sections were incubated with streptavidin‐conjugated HRP complex, followed by the addition of diaminobenzidine (Sigma‐Aldrich) and counterstaining with Mayer’s hematoxylin. Control experiments were performed by omitting the primary antibody or replacing it with the corresponding nonimmune serum.

### 2.9 Statistical analysis

Data were presented as mean ± *SD* of three separate experiments. Results were analyzed by analysis of variance for three groups or by Wilcoxon’s rank sum test between two groups. A *P* value of less than 0.05 was considered significant.

## 3 RESULTS

### 3.1 Short‐term incubation of PA increases Prx1, Prx2, and GPx1 protein expression in cultured podocytes

**Figure 1:**
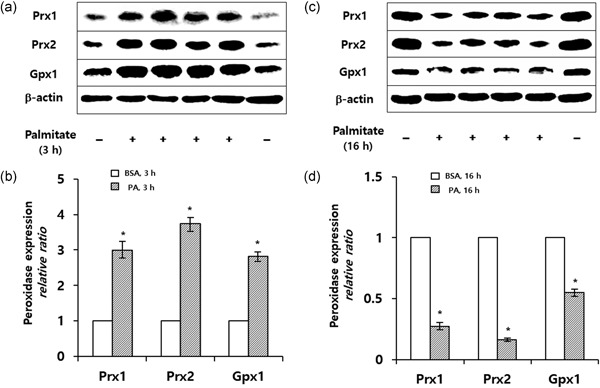
Immunoblots showing expression of Prx1, Prx2, and GPx1 in podocytes in response to 250 µM PA. (a) Three hours following PA exposure, levels of Prx1, Prx2, and GPx1 proteins are significantly increased as compared with controls. (b) Quantitative analysis of Prx1, Prx2, and GPx1 from three experiments is shown. (c) Sixteen hours following PA exposure, the levels of these proteins are significantly decreased as compared with controls. (d) Quantitative analysis of Prx1, Prx2, and GPx1 from three experiments is shown. Results are mean ± *SD* (*P < 0.05 vs. control). BSA, bovine serum albumin; GPx, glutathione peroxidase; PA, palmitic acid; Prx, peroxiredoxin

Incubation of podocytes with 250 µM PA for 1 hr showed no changes in Prx1, Prx2, and GPx1 expression as compared with controls. When cells were exposed to PA for 3 hr, the percentage of Prx1, Prx2, and GPx1 proteins was increased by 300 ± 71%, 375 ± 65%, and 283 ± 24%, respectively, as compared with controls (Figure 1a,b).

### 3.2 Long‐term incubation of PA decreases the expression of Prx1, Prx2, and GPx1 proteins in podocytes

After 6–8‐hr incubation with PA, the levels of Prx1, Prx2, and GPx1 proteins in podocytes began to decrease, reaching those of controls. At 16‐hr incubation with PA, these protein levels became significantly reduced by 0.28 ± 0.1, 0.16 ± 0.1, and 0.55 ± 0.1, respectively, as compared with controls (Figure 1c,d).

### 3.3 Long‐term incubation of PA reduces catalase expression in podocytes

**Figure 2:**
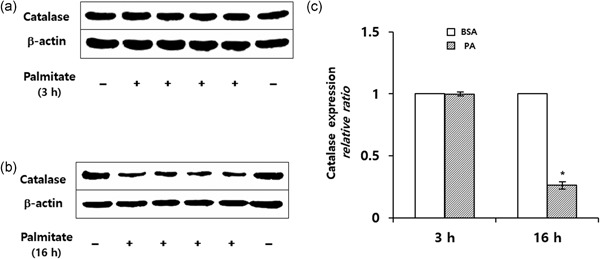
Immunoblots showing expression of catalase in podocytes in response to 250 µM PA. (a) Incubation of podocytes with PA for 3 hr induced no significant changes in the catalase protein expression as compared with controls. Sixteen hours following PA exposure, the catalase levels are significantly decreased as compared with controls. (c) Quantitative analysis of catalase from three experiments is shown. Results are mean ± *SD* (*P < 0.05 vs. control). BSA, bovine serum albumin; PA, palmitic acid

Incubation of podocytes with 250 µM PA for 3 hr induced no significant changes in catalase expression in podocytes (Figure 2a). After 16 hr, PA significantly decreased the catalase protein levels by 0.27 ± 0.03 as compared with controls (Figure 2b,c). Furthermore, catalase activity was decreased about 71% (0.33 ± 0.12 vs. 1.14 ± 0.54 µmol·mg protein^−1^·min^−1^; *P* < 0.05).

**Figure 3:**
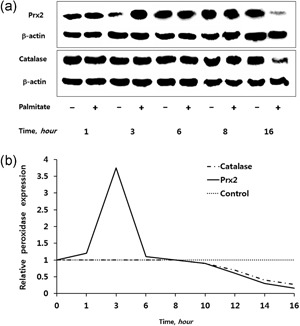
(a) Immunoblots showing sequential expression of Prx2 and catalase in podocytes incubated with PA for 1–16 hr. (b) Quantitative expression of Prx2 and catalase protein expression after correcting for the β‐actin signal. The Prx2 and catalase protein levels of PA‐treated cells are expressed as relative increases or decreases above or below the protein levels of untreated controls. PA, palmitic acid; Prx, peroxiredoxin

The time‐dependent effects of PA on Prx2 and catalase expression in podocytes are summarized in Figure 3.

### 3.4 OA restores the PA‐induced decreased peroxidase levels in podocytes

**Figure 4:**
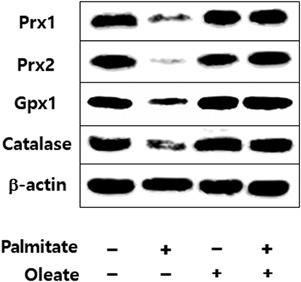
Immunoblotes showing coincubation of PA‐treated cells with 250 µM oleic acid for 16 hr restored the PA‐induced decreased Prx1, Prx2, GPx1, and catalase levels to controls. GPx, glutathione peroxidase; PA, palmitic acid; Prx, peroxiredoxin

Incubation of cells with 250 µM OA for 16 hr induced no significant changes in Prx1, Prx2, GPx1, and catalase protein expression as compared to controls, whereas PA markedly reduced the expression of these proteins. Coincubation of PA and OA in cultured podocytes for 16 hr restored the PA‐induced decreased Prx1, Prx2, GPx1, and catalase levels to those of controls (Figure 4).

### 3.5 H_**2**_O_**2**_ or HG induces no changes in catalase expression in podocytes

**Figure 5:**
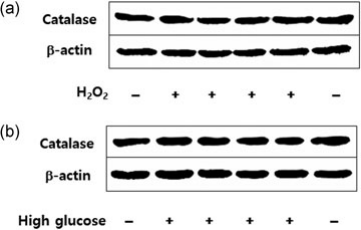
Immunoblot analysis showing expression of catalase in podocytes after 1 hr of incubation with 5 mM hydrogen peroxide (H_2_O_2_; a) and 72 hr of incubation with high glucose (25 mM; b)

When cultured podocytes were exposed to 3–5 mM H_2_O_2_ for 10 min to 1 hr, no significant difference was shown in catalase protein levels as compared to controls (Figure 5a). In addition, incubation of cells with HG (25 mM) for 24–96 hr induced no significant changes in catalase expression, either (Figure 5b).

### 3.6 ROS–H_**2**_O_**2**_ production is increased in the glomeruli of human DN

**Figure 6:**
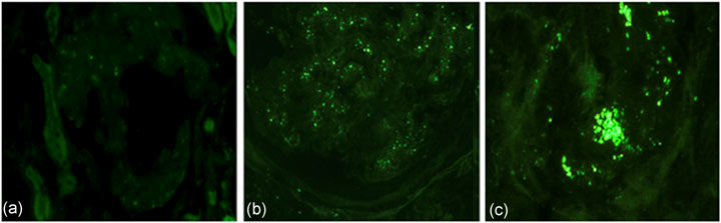
Frozen biopsy samples of control (a) and diabetic nephropathy (DN; b,c) exposed to carboxy‐DCFH DA‐AM for 2.5 hr. In control glomerulus, there is no DCF fluorescence (a). In DN, there is scattered DCF fluorescence in the glomerulus (b). In advanced DN, particularly, intraglomerular DCF fluorescence is markedly increased, forming patchy aggregates or clumps (c). Carboxy‐DCFH DA‐AM, carboxy‐2′,7′‐dichlorodihydrofluorescein diacetate diacetoxymethyl ester; DCF, dichlorofluorescein

In the controls, no DCF fluorescence was detected (Figure 6a). In DN, scattered DCF fluorescence appeared in the glomeruli, as shown by fluorescence microscopy (Figure 6b). In advanced DN, particularly, intraglomerular DCF fluorescence was markedly increased, even forming aggregates or clumps (Figure 6c).

### 3.7 Immunostaining for Prx1, Prx2, GPx1, and catalase is increased in the podocytes of advanced human DN

**Figure 7:**
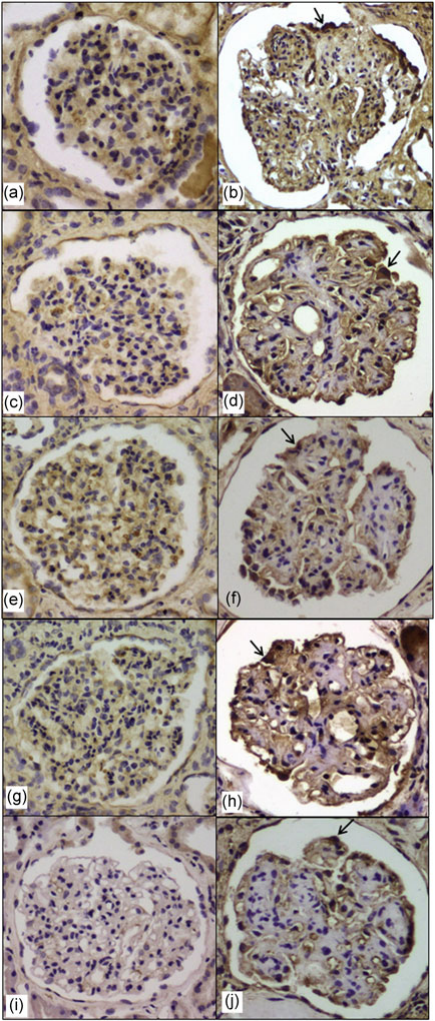
Immunostaining for peroxidases and TGF‐β1. In controls, there is no staining for Prx1 (a), Prx2 (c), GPx1 (e), catalase (g), and TGF‐β1 (i). In the cases of advanced diabetic nephropathy, there is strong immunostaining for Prx1 (b), Prx2 (d), GPx1 (f), catalase (h), and TGF‐β1 (j) in the podocytes mainly overlying the lesions of nodular sclerosis (arrows). Magnification: ×200. GPx, glutathione peroxidase; Prx, peroxiredoxin; TGF‐β1, transforming growth factor‐β1

In the controls, there was no glomerular staining for Prx1, Prx2, GPx1, catalase, and TGF‐β1 (Figure 7a,c,e,g,i). In the glomeruli of patients in the early stages of DN showing only slight mesangial expansion, immunostaining for these proteins was almost negligible. In the glomeruli of patients with advanced DN, there was moderate to strong staining for Prx1, Prx2, GPx1, and catalase in the podocytes, particularly in the glomeruli having the lesions of nodular sclerosis (Kimmelstiel–Wilson lesion; Figure 7b,d,f,h). TGF‐β1 immunostaining was also positive, the occurrence and distribution of which were similar to those of the peroxidase proteins (Figure 7j). In patients with idiopathic FSGS and moderately advanced IgAN, peroxidase proteins and TGF‐β1 were also expressed in the podocytes covering the segmentally sclerotic glomerular lesions.

**Figure 8:**
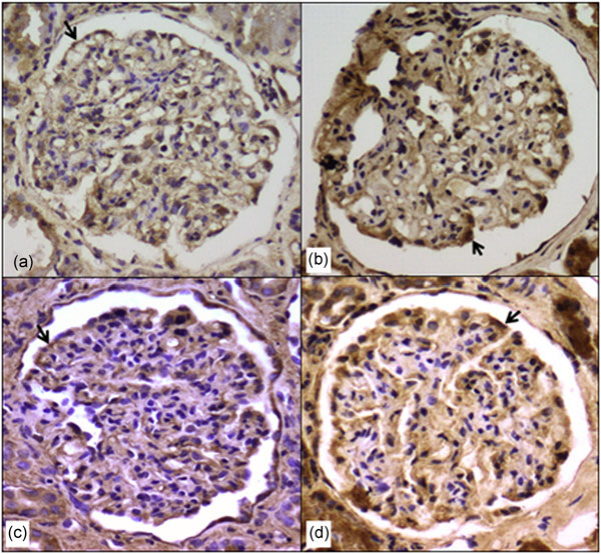
Immunostaining for peroxidases in moderately advanced diabetic nephropathy (a, b), FSGS (c), and IgAN (d). There is immunostaining for Prx1 (a), Prx2 (b), GPx1 (c), and catalase (d) in the podocytes of nonsclerotic glomeruli (arrows). Magnification: ×200. FSGS, focal segmental glomerulosclerosis; GPx, glutathione peroxidase; IgAN, IgA nephropathy; Prx, peroxiredoxin

Positive staining for Prx1, Prx2, GPx1, and catalase was occasionally observed in the podocytes of nonsclerotic glomeruli in the cases with moderately advanced DN (Figure 8a,b), FSGS (Figure 8c), or IgAN (Figure 8d).

When the slides were incubated with rabbit IgG (used as a negative control) instead of the primary antibody, no nonspecific staining was observed.

## 4 DISCUSSION

This study of the effects of PA on podocyte peroxidase expression had three main findings. First, PA induced an early transient increase in Prx1, Prx2, and GPx1 protein levels in cultured podocytes, but not catalase. Second, long‐term exposure of podocytes to PA decreased the expression of catalase, Prx1, Prx2, and GPx1 proteins. Third, DCF fluorescence was markedly increased in the advanced human diabetic glomeruli, together with an enhanced expression of Prx1, Prx2, GPx1, and catalase in the podocytes.

In this study, short‐term incubation of PA briefly increased the Prx1, Prx2, and GPx1 levels in podocytes. Recently, we showed that PA led to a significant increase in superoxide and H_2_O_2_ production in cultured podocytes (E. Lee et al., 2017). Thus, the enhanced expression of these peroxidase proteins could be the most feasible early response of podocytes to the intracellular ROS generation. In experimental DN, urinary excretion of Prx1 was also increased before development of overt histological damage (Korrapati et al., 2012).

Our demonstration of early transient elevation of Prx1 and Prx2 by PA supports the previous notion that Prxs, particularly Prx2, must react sufficiently rapidly with H_2_O_2_ to compete with other peroxidases, consuming basal levels of H_2_O_2_ (Peskin et al., 2007). Yet when peroxide production increases, Prxs are inactivated by peroxide‐induced hyperoxidation to protect the cell from further oxidative protein damage (Day et al., 2012). Indeed, we found that after 16‐hr incubation with PA, protein levels of Prx1 and Prx2 were decreased, which could be related to exhaustion of cytosolic Prx defense.

After Prxs are inactivated, catalase would then play a role to remove excess H_2_O_2_. The concerted role for Prx2 and catalase was demonstrated in tubular epithelial cell protection (Ruggiero et al., 2014). Nonetheless, incubation of podocytes with PA for 16–24 hr rather decreased the catalase levels in this study. Furthermore, neither HG nor 5 mM H_2_O_2_ enhanced podocyte catalase expression. Although Piwkowska et al. (2011) observed a decreased catalase protein expression after a 5‐day incubation of HG with podocytes, a maximum 4‐day incubation of HG in this study did not induce any changes in catalase expression. We could not extend the incubation time to 5 days because of severe cell apoptosis or lysis, not confirming their results.

High concentrations of exogenous H_2_O_2_ are required to elevate intracellular H_2_O_2_, because catalase rapidly breaks down H_2_O_2_ that enters the cells. Exogenous H_2_O_2_ only at very high concentrations, such as 3 mM, elevated intracellular H_2_O_2_ in distal nephron cells (Ma, 2011). Although we found no change in podocyte catalase expression by H_2_O_2_, others noted it either decreased (Y.‐H. Chen, Lin, Liu, & Su, 2017) or increased (Lu et al., 2015). Interestingly, patients with catalase gene mutation have life‐long increased H_2_O_2_ concentration, which has cytotoxic effects on pancreatic cells, to be a risk factor for diabetes (Goth, 2008).

We also observed that the decreased levels of Prx1, Prx2, GPx1, and catalase proteins in podocytes after long‐term PA exposure were restored by OA, supporting the previous notion that PA suppresses the antioxidative defense, whereas OA preserves it (Sargsyan et al., 2016).

To sum up, long‐term exposure of podocytes to PA decreased Prx1, Prx2, GPx1, and catalase levels. Thus, podocytes appear to be susceptible to PA‐induced oxidative damage with inadequate peroxidase activity.

Another important finding in this study is that the DCF signal is increased in the glomeruli of human DN, which is particularly severe in the late stages.

Mitochondria‐derived superoxide is rapidly dismutated to H_2_O_2_ through manganese superoxide dismutase. DCFH is used widely to detect intracellular H_2_O_2_, although it can react with a variety of other cellular oxidants besides H_2_O_2_ (Rhee, Chang, Jeong, & Kang, 2010). Thus, an enhanced DCF fluorescence signal in the diabetic glomeruli in this study can represent the increased ROS–H_2_O_2_ dismutated from the mitochondrial superoxide. In support of this notion, Galvan et al. (2017) demonstrated an increased mitochondrial ROS generation in the glomeruli of living diabetic mice. In addition, urinary H_2_O_2_ level is significantly elevated in diabetic mice (Sharma et al., 2008), suggesting that increased H_2_O_2_ in diabetic glomeruli might be an important source of H_2_O_2_ in urine.

In the current study, there was strong immunostaining for Prx1, Prx2, GPx1, and catalase in the podocytes of patients with late stages of DN, FSGS, and IgAN. These proteins were also focally expressed in the podocytes of nonsclerotic glomeruli in the moderate stages of nephropathies, suggesting that their expression is not confined to the advanced stages of disease. Furthermore, our findings suggest that the occurrence of peroxidases on renal biopsies at the early stages of nephropathies predicts a progressive nature of the disease with sclerotic glomerular lesions, which are not detected by sampling problems. The increased expression of peroxidase proteins in the podocytes of diabetic kidneys appears to contradict our in vitro study, the mechanisms of which are not clear.

TGF‐β is produced as latent complexes. Unlike mesangial cells, podocytes do not secrete TGF‐β in response to common in vitro fibrogenic stimuli (S. Chen et al., 2005; Iglesias‐de la Cruz et al., 2002). As yet mesangial immunostaining for active TGF‐β1 is frequently negative in chronic glomerular disease (J. H. Kim, Kim, Moon, Hong, & Lee, 2003; H. W. Kim, Moon, Park, Hong, & Lee, 2002; Wahab et al., 2005), while podocytes covering the sclerotic segments exhibit increased expression of TGF‐β1 protein as shown in the current study. In this regard, H. S. Lee and Song (2009) suggested that TGF‐β secreted as latent complexes by mesangial cells is stored in mesangial matrix in chronic glomerular disease, from which soluble forms of latent TGF‐β are released and localized to the podocyte surface. Podocyte‐derived ROS seem to be involved in TGF‐β activation in podocytes. In this study, TGF‐β1 immunostaining was also positive in the podocytes of advanced DN, wherein overexpression of peroxidases was shown. The coexpression of TGF‐β1 and peroxidases in the current study suggests that the peroxidases could not suppress the activation of latent TGF‐β localized to the podocytes of diabetic kidneys. Rather, possibly transformed podocytes in advanced DN by TGF‐β‐induced epithelial‐to‐mesenchymal transition (H. S. Lee, 2012; Loeffler & Wolf, 2015) might overexpress peroxidases in response to excess ROS generation. The marked ROS generation by severe long‐term podocyte injury seems to surpass the cellular antioxidant defense capacity of peroxidases. Thus, despite their overexpression, peroxidases may have only futile antioxidant effects in the podocytes of advanced DN.

In summary, long‐term exposure of cultured podocytes to PA significantly decreased the protein levels of peroxidases, whereas immunostaining for peroxidases and TGF‐β1 was increased in the podocytes in the late stages of DN. These findings suggest that podocytes are susceptible to PA‐induced oxidative damage with impaired peroxidase activity and that peroxidases have only futile antioxidant effects in the podocytes of advanced DN. Thus, PA‐induced podocyte injury via inadequate peroxidase response to H_2_O_2_ appears to play an important role in the pathogenesis of DN.

## CONFLICTS OF INTEREST

The authors report no conflicts of interest.

## AUTHORS’ CONTRIBUTIONS

H.S.L. provided the conception and design of research. H.S.L., and E.L. performed experiments, analyzed data, and interpreted the results of experiments. E.L. prepared figures. H.S.L. wrote the manuscript.
